# Expanding the genetic programmability of *Lactiplantibacillus plantarum*

**DOI:** 10.1111/1751-7915.14335

**Published:** 2023-08-28

**Authors:** Marc Blanch‐Asensio, Sourik Dey, Varun Sai Tadimarri, Shrikrishnan Sankaran

**Affiliations:** ^1^ Bioprogrammable Materials, INM—Leibniz Institute for New Materials Saarbrücken Germany

## Abstract

Lactobacilli are ubiquitous in nature and symbiotically provide health benefits for countless organisms including humans, animals and plants. They are vital for the fermented food industry and are being extensively explored for healthcare applications. For all these reasons, there is considerable interest in enhancing and controlling their capabilities through the engineering of genetic modules and circuits. One of the most robust and reliable microbial chassis for these synthetic biology applications is the widely used *Lactiplantibacillus plantarum* species. However, the genetic toolkit needed to advance its applicability remains poorly equipped. This mini‐review highlights the genetic parts that have been discovered to achieve food‐grade recombinant protein production and speculates on lessons learned from these studies for *L. plantarum* engineering. Furthermore, strategies to identify, create and optimize genetic parts for real‐time regulation of gene expression and enhancement of biosafety are also suggested.

## Graphical abstract

Lactobacilli are ubiquitous in and around us, providing considerable benefits for food processing and healthcare. There is growing interest to enhance their capabilities through genetic engineering and *Lactiplantibacillus plantarum* is the most extensively explored for this. This mini‐review emphasizes the need for expanding the genetic toolkit of *L. plantarum* and describes strategies to optimize its gene expression and biosafety.

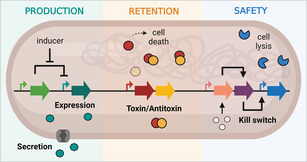

## INTRODUCTION

**FIGURE 1:**
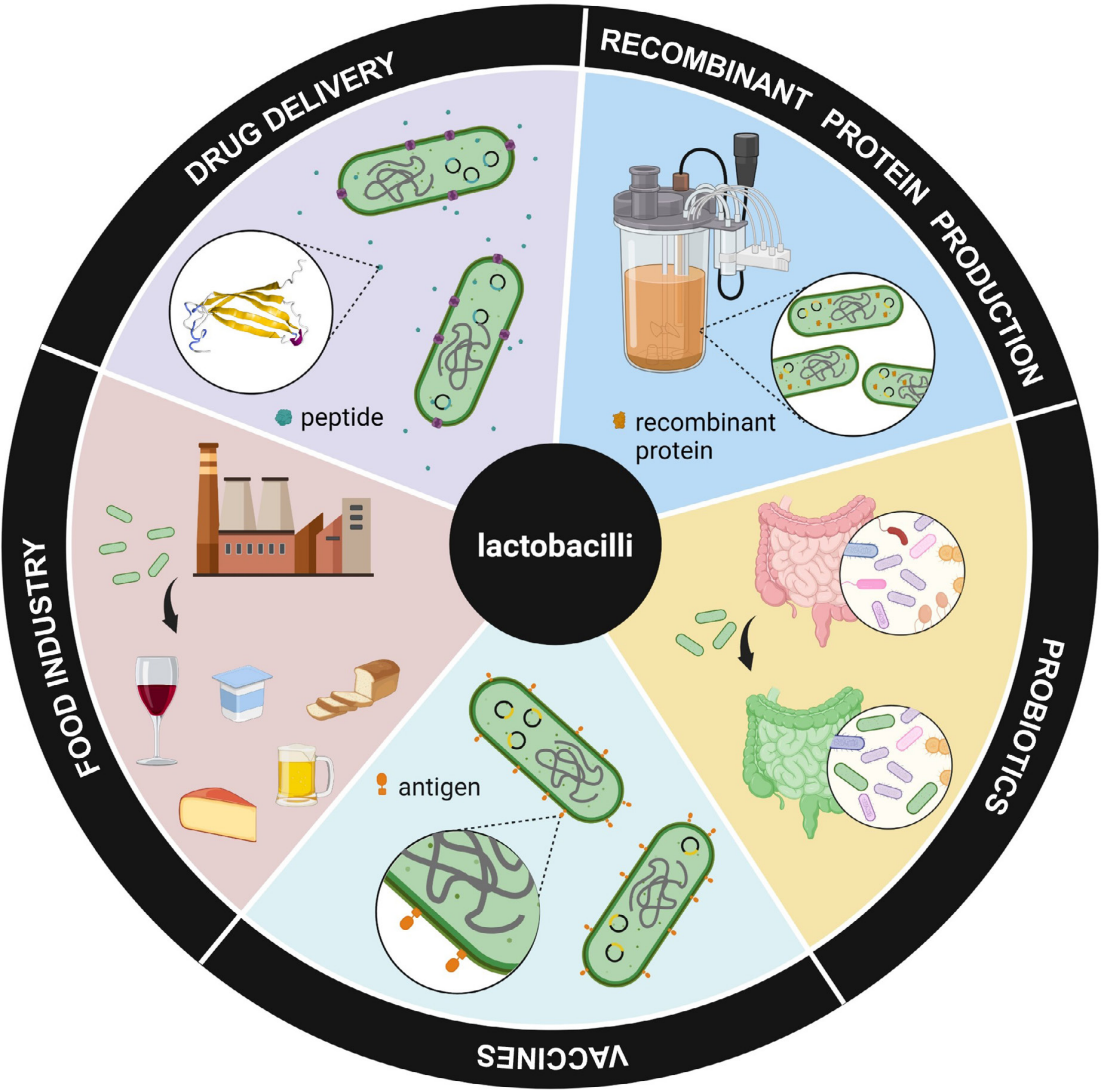
Schematic showing the potential applications of genetically engineered lactobacilli in drug delivery, food fermentation, vaccine development, probiotic enhancement and recombinant protein production.

In recent decades, advancements in bacterial synthetic biology have opened more doors to bacteria‐based applications beyond bioprocessing, like bioremediation, biosensing and living therapeutics. Among the many diverse types of bacteria that exist in nature, lactobacilli occupy a central beneficial role for humans, supporting human, animal and plant health as commensals and probiotics (Di Cerbo et al., 2016) and driving fermentation in the food industry (Dewi & Kollanoor Johny, 2022). Due to this, there has been considerable interest in genetically programming these bacteria for enhancing their role as probiotics, increasing pharmaceutical protein production and establishing their role as living drug delivery vectors and mucosal vaccine candidates (Wu et al., 2021). The application fields where genetically engineered lactobacilli play a very important role have been highlighted in Figure 1. This is in line with the growing development of several lactic acid bacteria (e.g. lactococci, pediococci, streptococci etc.) for such applications through the steady expansion of their genetic toolboxes (Bosma et al., 2017; Landete, 2017; Plavec & Berlec, 2020; Wu et al., 2021).

**FIGURE 2:**
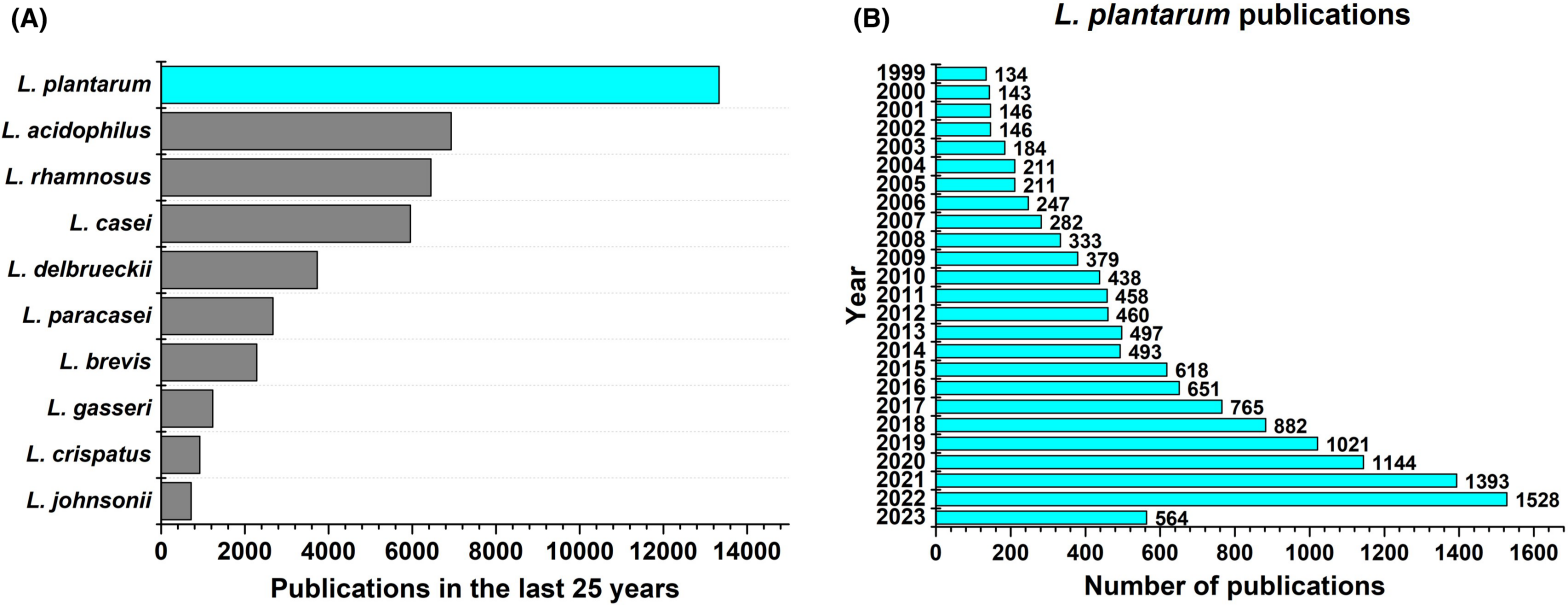
(A) Publication counts of different pre‐2020 *Lactobacillus* species over the last 25 years. (B). Yearly publication counts of *Lactiplantibacillus plantarum* from 1999 to 2023 (June). The statistics were gathered using the Web of Science by performing topic searches for each species using their pre‐2020, post‐2020 and abbreviated phylogenetic names.

Due to steady growth in the variety of species discovered as lactobacilli, this genus was split into 25 genera in 2020 based on whole genome sequences (Zheng et al., 2020). Among this vast variety, *Lactiplantibacillus plantarum* is the most extensively studied and engineered species as exemplified by the number of publications in the last 25 years (Figure 2A) and an increasing number of reports to date (Figure 2B).

This is due to a combination of multiple reasons such as:

- *Lactiplantibacillus plantarum* strains have been qualified with a ‘generally recognized as safe’ (GRAS) status by the US Food and Drug Administration (USFDA).
- They have proven to be effective probiotics for human health with beneficial effects such as lowering cholesterol levels (Naruszewicz et al., 2002), attenuation of inflammatory bowel diseases (Schultz et al., 2002) and prevention of *Clostridium difficile* infections (Kujawa‐Szewieczek et al., 2015).
- They are extensively used in the food industry as natural alternatives for chemical additives in dairy products (Arena et al., 2016) and as supplements to increase the shelf‐life of fermented food products (Behera et al., 2018).
- They are among the few lactobacilli species that are considered to be nomadic in nature, capable of occupying a wide variety of niches (Duar et al., 2017). For instance, they have been found to thrive in the oral cavity, guts and vaginas of vertebrates, in the guts of fish and insects, in dairy products, fermented fruits and vegetables, etc. That is because they encode diverse metabolic pathways, like those for uptake and utilization of a large spectrum of sugars as carbon sources, for exopolysaccharide synthesis and protein secretion. Also, unlike many other lactobacilli species, they can synthesize most amino acids and are auxotrophic to only a few, namely valine, leucine, isoleucine and glutamate.
- Most strains have high‐stress tolerance to acid, alkaline and osmotic stresses, while some are additionally tolerant to heat, oxidative and starvation stresses (Parente et al., 2010). Due to this, they can survive in gastric juices and bile acids, apart from diverse environmental conditions.
- They are facultatively anaerobic or microaerophilic and can be easily cultured in the lab and in bioreactors.
- The genomes of multiple strains such as WCFS1, JDM1, NC8, ST‐III and many more have been sequenced, leading to an improved understanding of their metabolic, biochemical and physical characteristics (Siezen & van Hylckama Vlieg, 2011).
- They are genetically tractable and several genetic parts including plasmid replicons, promoters, signal peptides for secretion, plasmid retention systems (antibiotic resistance, auxotrophic complementation, toxin–antitoxin) and gene editing tools have been demonstrated to work reliably and stably (Zhou et al., 2019).
- Inducible gene expression has been demonstrated using the class II bacteriocin sakacin peptide‐inducible two‐component system (TCS) (from *Latilactobacillus sakei*; Sørvig, Mathiesen, et al., 2005; Sørvig, Skaugen, et al., 2005). This, along with their protein secretion capability, has led to the exploration of *L. plantarum* strains for the heterologous expression of recombinant proteins.

Multiple genetic parts developed in *L. plantarum* have been shown to function across other lactobacilli genera and species (Sørvig et al., 2003). Thus, *L. plantarum* has been used as a gateway host for developing cross‐genera compatible genetic parts.

Despite these advances, the synthetic biology toolbox of *L. plantarum* pales in comparison to model microbes like *Escherichia coli*, *Bacillus subtilis* or *Lactococcus lactis*. This is exemplified by the lack of strong promoters, reliable repressors and orthogonal polymerases. Such tools are essential for the development of genetic circuits in *L. plantarum* and lactobacilli as a whole, which will allow them to be programmed with enhanced functions for various applications as already described.

In this minireview, we have addressed this technological gap and suggested unexplored or poorly explored options to fill it. We cover four crucial aspects concerning the genetic programmability of *L. plantarum* and the possibility to establish this species as a model *Lactobacillus* chassis for synthetic biology applications. The first part covers gene expression modules, with a focus on existing and potential genetic parts that enable tuning or regulation of gene expression in *L. plantarum*. The second part highlights advances in programming protein secretion from *L. plantarum* and offers strategies to accelerate signal peptide screening. The third part involves plasmid bioretention, particularly focusing on approaches that do not require antibiotic selection pressures. In the last part, we cover biocontainment strategies to ensure the programmable death of these genetically modified bacteria and prevent their release into the environment.

### Tuning and regulation of gene expression

Promotion and control of gene expression can be encoded and tuned using promoters, repressors, ribosome binding site (RBS) sequences, RNA polymerases, sigma factors etc. The greatest advances in *L. plantarum* have been in the development of constitutive and inducible gene expression systems through promoter screening, the adaptation of TCSs and repressor engineering attempts. These aspects have been briefly covered in this section, followed by suggestions to identify new parts.

## CONSTITUTIVE GENE EXPRESSION SYSTEMS

A repertoire of promoters is crucial for establishing synthetic genetic circuits in a microbial chassis (Mays & Nair, 2018; Mugwanda et al., 2023). Different strategies have been pursued to increase the available set of promoters for *L. plantarum*, like screening native promoters from the bacterial genome, creating synthetic promoter libraries by random or directed mutagenesis and employing promoter regions from both phylogenetically related and distant bacterial species. From the diverse set of native promoters tested in *L. plantarum*, P_ldh_ (Anbazhagan et al., 2013; Reveneau et al., 2002) and P_tuf_ (Spangler, Caruana, et al., 2019; Spangler, Dean, et al., 2019) have been used most frequently by researchers. Comparative studies have shown that these two promoters can drive the expression of heterologous genes at moderately high levels in the bacterial chassis (Peirotén & Landete, 2020). However, endogenous promoters can also be affected by native metabolic networks during cellular growth and division, which can affect the overall expression of a heterologous protein (Wang, Fu, et al., 2020; Wang, Liang, et al., 2020). To bypass promoter cross‐reactivity and improve the overall transcription rate, synthetic promoter libraries were created and tested in *L. plantarum*. The first reported synthetic promoter library used the 16S rRNA promoter template to create an array of artificial promoters of varying degrees of strength in *L. plantarum* WCFS1 (Rud et al., 2006). Promoters P_48_ and P_11_ from this library showed the highest gene expression levels for multiple heterologous protein‐encoding genes (Guo et al., 2019; Ma et al., 2016). Apart from native promoter templates, the constitutive P_23_ promoter of *L. lactis* was rationally mutagenized to create a promoter library of different strengths. From this library, the P_OL2_ promoter showed the highest expression strength in the *L. plantarum* strains XJ25, XJ14 and XJA2 isolated from Chinese red wine and traditional Chinese pickle (Meng et al., 2021). However, these reports relied on saturation mutagenesis of spacer regions in promoter sequences which predominantly leads to decreased promoter strength (Blazeck & Alper, 2013). To increase the overall transcription rate, efforts are being made to develop hybrid promoters with potent enhancer elements fused to conserved core promoter regions as successfully demonstrated in unicellular yeasts (Cazier & Blazeck, 2021). Apart from the transcription rate, the translational efficiency of heterologous proteins has also been seen to be affected by spacer lengths between the RBS and the start codon (Tauer et al., 2014). Significant downregulation in the protein yield is expected when the spacer length is not in the range of 7–11 base pairs (bp). Yet, it is still unclear whether the nucleotide composition of the spacer also plays a role in regulating the protein yield.

Promoters from non‐endogenous sources have also shown considerable potential for protein production in *L. plantarum*, *L. lactis* and *L. acidophilus*, and have been extensively used for orthogonal promoter analysis from phylogenetically related bacteria. These isolated promoters have played an important role in enhancing the performance of *L. plantarum* in industrially relevant processes. For example, the strong lactococcal promoter P_59_ was used for the recombinant expression of catalase in *L. plantarum* TISTR850, which helped significantly reduce the lipid oxidation level in fermented meat (Noonpakdee et al., 2004). The P_32_ promoter from *L. lactis* was used for the overexpression of the stress response regulator protein (ctsR) to increase the ethanol tolerance of *L. plantarum* WCFS1 during wine fermentation (Zhao et al., 2019). Promoters P_pgm_ and P_SlpA_ isolated from *L. acidophilus* strains were used for surface anchoring of the β‐mannanase protein in *L. plantarum*, which showed enzyme activities of 1200 and 3500 U per gram of biomass (Nguyen et al., 2019).

Apart from related genera, constitutive promoters from phylogenetically distant bacteria have been also tested in *L. plantarum* strains. The constitutive promoter P_X_, isolated from the Gram‐positive bacteria *Streptococcus pneumoniae*, was able to drive high expression of the red fluorescent (mRFP5) protein in probiotic *L. plantarum* 90 and B2 strains (Russo et al., 2015). The strong fluorescent protein expression allowed monitoring of bacterial colonization in the intestinal tract of zebrafish larvae. The P_X_ promoter‐driven mRFP5 expression also helped assess the stress tolerance and adhesion capability of the riboflavin overproducer *L. plantarum* M5MA1‐B2 strain in the mouse digestive tract (Mohedano et al., 2019). In line with these findings, alternate promoters from Gram‐negative bacteria were tested in *L. plantarum* WCFS1 (Dey et al., 2023). Although the promoters P_R_ and P_L_ (strong promoters reported in *E. coli*) gave very low levels of fluorescent protein expression, the promoter P_tlpA_ (strong promoter in *Salmonella typhimurium*) surpassed the protein expression levels of previously described strong promoters like P_48_ and P_23_ by fivefold. Although the reason for the high expression strength is still unknown, it has previously been reported that the rpoD RNA polymerase of *L. plantarum* has an innate ability to recognize conserved regions of orthogonal promoters and initiate mRNA production irrespective of the expression host (Gaida et al., 2015).

These studies have yielded a collection of well‐characterized constitutive promoters with strengths varying across several orders of magnitude, enabling tuning of protein expression levels (Table 1). However, even the strongest constitutive promoters in *L. plantarum* are weaker than inducible promoters in *E. coli*. One key disadvantage in increasing constitutive promoter strengths is that protein expression competes with the natural metabolism in the cell and decelerates growth. Inducible promoters help circumvent this condition by enabling temporal separation of the growth and production phases, thereby allowing overexpression to occur only in well‐grown healthy biomass. The next section covers examples of such inducible gene expression systems in *L. plantarum*.

**TABLE 1:** Examples of constitutive gene expression systems tested in *Lactiplantibacillus plantarum*.

Origin strain	Promoter	Relative expression strength^a^	Reference
*Constitutive expression systems*			
*Lactiplantibacillus plantarum*	P_ldh_	—	Reveneau et al. (2002)
*Lactiplantibacillus plantarum*	P_tuf_	3	Spangler, Caruana, et al. (2019), Spangler, Dean, et al. (2019)
*Lactiplantibacillus plantarum*	P_48_	3	Rud et al. (2006)
*Lactiplantibacillus plantarum*	P_11_	3	Rud et al. (2006)
*Lactiplantibacillus plantarum*	P_21_	2	Rud et al. (2006)
*Lactiplantibacillus plantarum*	P_44_	1	Rud et al. (2006)
*Lactiplantibacillus plantarum*	P_23_	3	Meng et al. (2021)
*Lactococcus lactis*	P_OL1_	2	Meng et al. (2021)
*Lactococcus lactis*	P_OL2_	3	Meng et al. (2021)
*Lactococcus lactis*	P_OL3_	1	Meng et al. (2021)
*Lactococcus lactis*	P_69_	—	Noonpakdee et al. (2004)
*Lactococcus lactis*	P_32_	—	Zhao et al. (2019)
*Lactobacillus acidophilus*	P_pgm_	—	Nguyen et al. (2019)
*Lactobacillus acidophilus*	P_slpA_	—	Nguyen et al. (2019)
*Streptococcus pneumoniae*	P_X_	—	Russo et al. (2015)
*Salmonella typhimurium*	P_tlpA_	5	Dey et al. (2023)

^a^ Expression strengths have been computed in relation to the PtlpA promoter based on comparative expression levels reported in the different studies. Since expression levels in different studies were tested under different conditions, these values are approximations on a scale from 1 to 5, with 5 being the highest expression level and each integer lower representing a twofold decrease. Rows without numbers were from studies where such relative expression strengths could not be estimated.

## INDUCIBLE GENE EXPRESSION SYSTEMS

**FIGURE 3:**
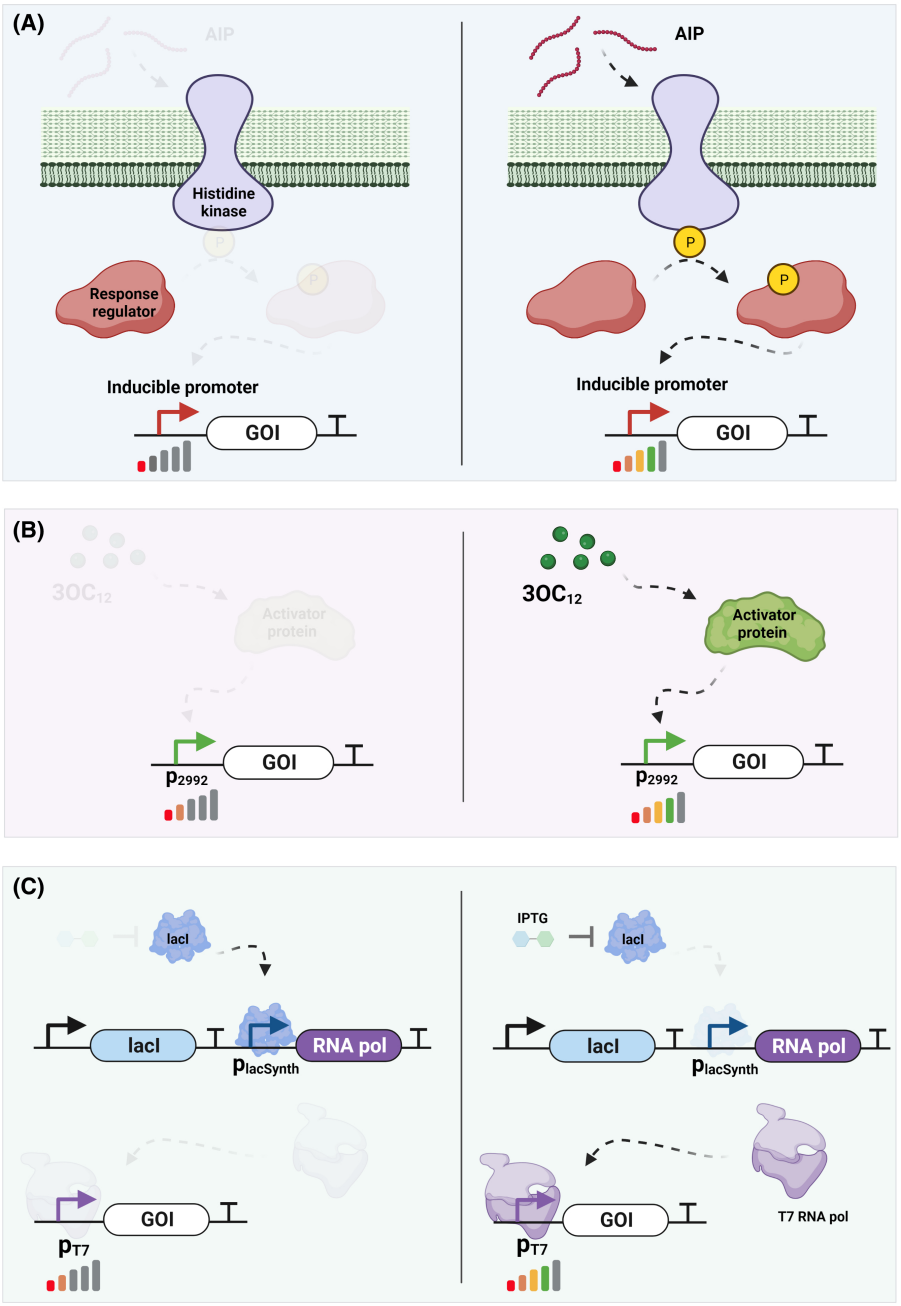
Scheme showing the inducible protein expression systems in *Lactiplantibacillus plantarum* (A) TCS pSIP system. The AIP interacts with the histidine kinase, which gets phosphorylated and transfers the phosphate to the response regulator protein. This protein then triggers the expression of the gene of interest (GOI) by activating the inducible promoter. (B) 3OC_12_ system. The addition of 3OC_12_ induces the expression of the GOI by activating the P_2992_ promoter through an unknown activator protein. (C) T7RNAP system. IPTG inhibits the lacI repressor, thus stopping the repression of P_lacSynth_ and promoting the production of the T7 RNA polymerase, which drives the expression of the GOI.

While constitutive promoters enable the tuneable expression of proteins, inducible systems provide temporal control of them. On the one hand, this is useful for optimizing bioprocess conditions to improve protein yields and on the other, it enables the construction of layered genetic circuits for more advanced applications (Wong et al., 2015). The most widely used inducible systems reproducibly verified in *L. plantarum* are based on pSIP vectors (Sørvig et al., 2003). These vectors encode TCSs systems that are originally part of quorum sensing‐based bacteriocin regulation operons in *L. sakei* and are induced by autoinducer peptides (AIP) (Figure 3A). With this AIP inducible system, several recombinant proteins have been expressed in a dose‐dependent manner in *L. plantarum* at decent yields. The pSIP411 vector produced 1800 Miller Unit equivalents (MU) of β‐glucuronidase (GusA) in *L. plantarum* NC8 post‐induction with 50 ng/mL sakacin inducer peptide (SppIp), accounting for a fold induction of 87. On the other hand, 50 ng/mL of SppIP induction for the pSIP407 vector produced the protein aminopeptidase‐N (PepN) at a specific activity of 3.5 U/mg of protein, constituting up to 40% of the total intracellular protein content of the bacterial host (Mathiesen et al., 2004; Sørvig, Mathiesen, et al., 2005; Sørvig, Skaugen, et al., 2005). Notably, the AIP inducible pSIP system has enabled both the extracellular secretion of recombinant proteins such as nuclease (Karlskås et al., 2014; Tran et al., 2021) as well as cell‐surface anchoring of proteins for applications ranging from biocatalysis (Nguyen et al., 2016) to mucosal vaccine development (Li et al., 2021; Wang, Fu, et al., 2020; Wang, Liang, et al., 2020). However, the heterologous protein yield can decrease significantly when AIP induction is conducted at growth phases other than the early exponential phase (personal communication). An alternative AIP inducible system commonly used in lactococci is based on the polycyclic inducer peptide, nisin and the nisK–nisR regulatory cascade (Mierau et al., 2005). This dual‐plasmid system was originally unsuitable for the expression of the tetanus toxin (TTFC) in *L. plantarum* WCFS1 (Pavan et al., 2000). However, when the nisK–nisR regulatory modules were integrated into the bacterial genome, the TTFC yield constituted about 10% of the total intracellular protein content post 25 ng/mL of nisin induction.

Transcriptomic and proteomics analysis showed that when *L. plantarum* WCFS1 cultures were subjected to treatment with N‐3‐oxododecanoyl homoserine lactone (3OC_12_), the predominant Acyl‐homoserine lactone (AHL) derived from *Pseudomonas aeruginosa*, the gene expression levels of luxS and plantaricin were significantly upregulated (Spangler, Caruana, et al., 2019; Spangler, Dean, et al., 2019). Detailed analysis of the upregulated gene networks showed that 3OC_12_ induction for 4 h was able to significantly increase the expression levels of promoters P_2992_ and P_3214_ (Spangler et al., 2022). A small set of promoters generated by modifying the −10 and −35 regions of the P_2992_ promoter resulted in a complete loss of 3OC_12_‐based induction, whereas the R8 mutant (8 bp spacer) retained the AHL induction ability even though the overall expression was severely compromised (~1000 fold). This study proved that the P_2992_ consensus promoter sequence can be a potential part of a TCS whose activation mechanisms are yet to be elucidated and expanded (Figure 3B).

In addition to AIP and AHL, natural sugars and related analogues have also been explored as inducer molecules for gene expression. An endogenous promoter (P_lacA_) system showed an ~8‐fold induction capability in response to 2% (w/v) lactose in *L. plantarum* 3NSH (Heiss et al., 2016). The system was completely repressed in the presence of monomeric sugars (glucose and galactose), indicating the presence of catabolite‐responsive control in the promoter region (Marasco et al., 1998). The orthogonal xylose inducible promoter (P_xylA_), derived from *Bacillus megaterium*, was responsive to xylose supplementation but showed significant leaky expression in the absence of the inducer, reducing the dynamic range to only ~2‐fold. This fold change was only achievable when the sole carbon source for bacterial growth was replaced from glucose to galactose. This study also tested the ability of T7 RNA Polymerase (T7 RNAP) to drive protein expression in *L. plantarum* 3NSH. The reporter mCherry gene was encoded downstream of the P_T7_ promoter in the high copy plasmid pCDLbu1 and the codon optimized T7RNAP was expressed by the isopropyl β‐D‐1‐thiogalactopyranoside (IPTG) inducible P_lacSynth_ promoter on a low copy pCD256 vector (Figure 3C). Post IPTG induction, the dynamic range of the system accounted for an ~6‐fold higher mCherry expression. The low induction fold changes of these systems were attributed to the weak promoters driving insufficient repressor protein expression, leading to a high basal level expression of reporter proteins. The maximum protein expression rate of all the inducible promoters showed ~6‐fold lower expression in comparison to the synthetic constitutive promoter P_11_. Rational modification of the lactose/galactose inducible P_lacA_ and P_lacLM_ promoters isolated from *L. plantarum* WCFS1 increased their expression strengths to ~10 fold and ~13 fold, respectively, in comparison to their native promoter sequences (Zhang et al., 2019). The β‐galactosidase yield (45.72 ± 0.44 U/mL) of the modified P_lacLM‐35‐10_ promoter was significantly higher than the well‐established expression systems driven by promoters P_spp_ and P_ldh_.

Several stress‐inducible systems, from metal starvation and bile stress to temperature fluctuations, have also been established in *L. plantarum*. The promoter P_MntH2_ showed an inverse relationship to manganese ion presence, producing β‐glucosidase (CelB) at an enzyme activity of ~18 per mg of total protein in the complete absence of MnSO4 (Böhmer et al., 2013). However, the overall protein yield was 60 times lower than the pSIP‐based inducible system, suggesting low expression strength of promoter P_MntH2_. Promoter P_16090_ isolated from *Lactobacillus casei* BL23 was tested for its bile stress induction capacity in other bacterial species and was observed to give the highest induction in *L. plantarum* WCFS1 (Martínez‐Fernández et al., 2019). The strain was able to survive at high bile salt concentrations (>0.2% w/v) and showed no leaky expression of the fluorescent reporter protein, evoglow‐Pp1. The promoter P_ldh_ originally encoding for the L‐lactate dehydrogenase enzyme in *Lactobacillus johnsonii* PF01 was also shown to be responsive to bile supplementation in *L. plantarum*, with an induction fold change of ~1.8 (Chae et al., 2019). Finally, a temperature gradient shift from 27 to 8°C has been shown to activate the cold shock response promoter, P_cspL_, and increase the expression of the reporter protein β‐galactosidase by ~1.4 fold (Derzelle et al., 2002).

**FIGURE 4:**
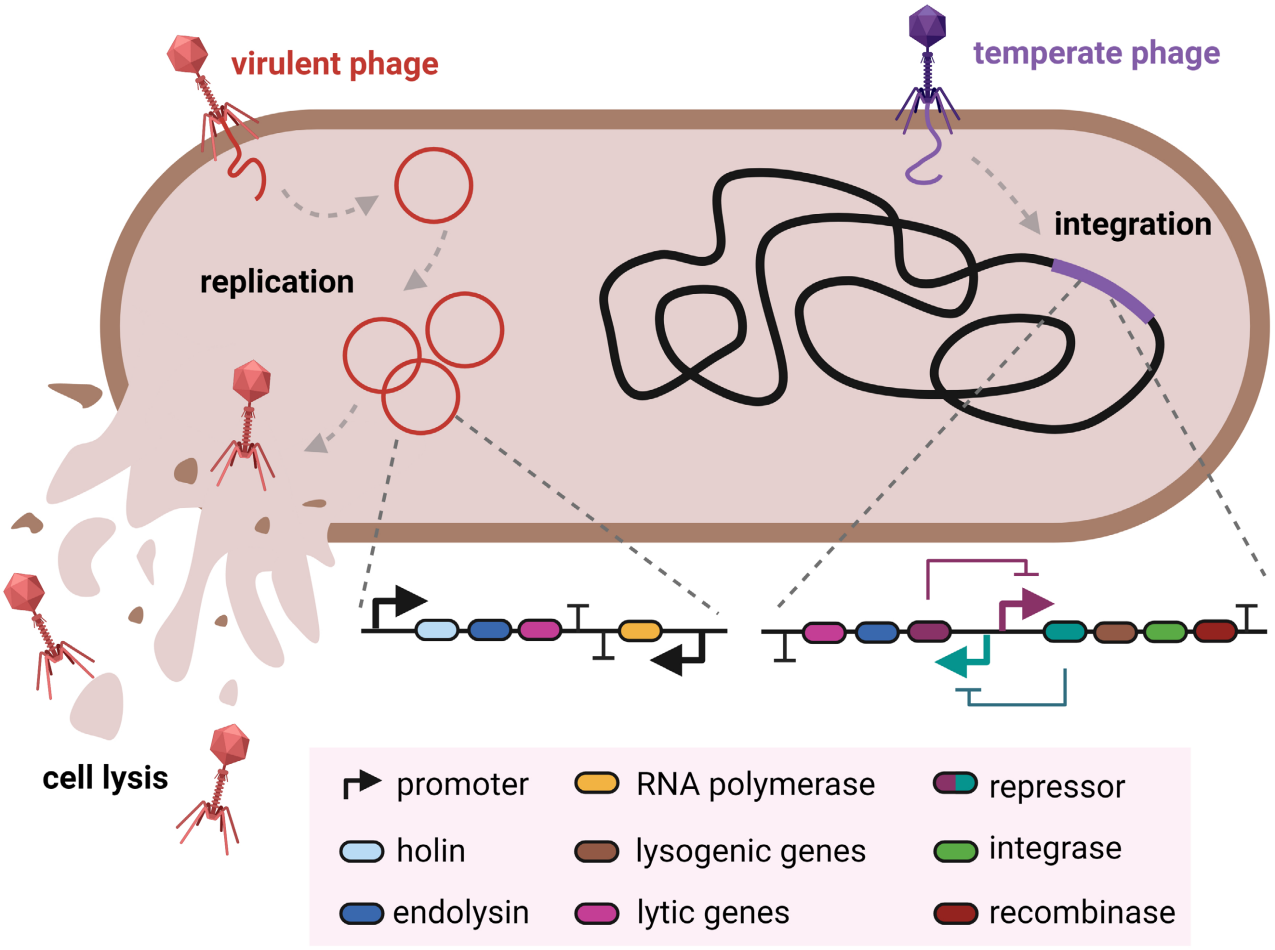
Schematic figure showing a bacterium being infected by both a virulent phage and a temperate phage. Relevant genes crucial for the lytic and the lysogenic cycles are highlighted in the genomes of the virulent and temperate phage respectively.

These few studies reveal that inducible protein expression (Table 2) in *L. plantarum* is severely limited, and additional robust and versatile systems are required. To develop such systems, genetic parts involved in gene regulation, like repressors, polymerases, etc., need to be identified. One crucially untapped source for gene regulatory parts includes bacteriophages that infect lactic acid bacteria. They naturally encode repressors in endogenous genetic switches that control the lysogenic and lytic cycles (Brady et al., 2021; Figure 4). Furthermore, phage‐derived genetic parts are naturally adapted to their hosts due to the arms race between bacteriophages and bacteria and millions of years of coevolution (Hampton et al., 2020).

**TABLE 2:** List of inducible gene expression systems tested in *Lactiplantibacillus plantarum*.

Origin species/strain	Promoter	Inducer	Dynamic range^a^	Reference
*Inducible expression systems*				
*Lactobacillus sakei*	P_sppA_	SppIp (AIP)	87	Mathiesen et al. (2004)
*Lactococcus lactis*	P_nisA_	Nisin (AIP)	20	Pavan et al. (2000)
*Lactiplantibacillus plantarum* WCFS1	P_2992_	3OC_12_ (AHL)	10	Spangler et al. (2022)
*Lactiplantibacillus plantarum* WCFS1	P_lacA_	Lactose	8	Heiss et al. (2016)
*Bacillus megaterium*	P_xylA_	Xylose	2	Heiss et al. (2016)
T7 bacteriophage	P_T7_	IPTG	6	Heiss et al. (2016)
*Lactiplantibacillus plantarum* WCFS1	P_lacLM_	Galactose	13	Zhang et al. (2019)
*Lactobacillus johnsonii* PF01	P_ldh_	Bile Salt	1.8	Chae et al. (2019)
*Lactiplantibacillus plantarum* NC8	P_cspL_	Cold Shock	1.4	Derzelle et al. (2002)

^a^ Dynamic ranges were calculated from data reported in each study as the ratio of protein expression level on induction by expression level without induction.

There are several publications based on the identification and characterization of repressors encoded in genetic switches of phages that infect *L. plantarum* and other species closely related to *L. plantarum* (Binishofer et al., 2002; Kakikawa et al., 2000; Ladero et al., 1998). However, there are no reports on the experimental testing of these repressors in *L. plantarum*. Exploring and implementing these repressors in novel genetic circuits in *L. plantarum* could be an interesting approach to expanding the genetic programmability of these bacteria. Besides, there is evidence that phage‐derived parts can be functional in *L. plantarum*. In 2015, Yang and colleagues employed phage recombinases encoded by a prophage within the genome of *L. plantarum* WCFS1 as a genetic tool to successfully manipulate the genome of these bacteria (Yang et al., 2015). Such recombinases are frequently found in the genomes of prophages since these are necessary to integrate the genome of the phage into the genome of the bacteria (Smith, 2015) and have extensively been used for recombineering, a technique for efficient genetic engineering (Li et al., 2023). If phage‐derived repressors prove to be effective in blocking the expression of the target gene in *L. plantarum*, they could be further modified to respond to external factors (e.g. temperature, light, chemicals), as it has previously been done for the Lambda phage repressor cI (Xiong et al., 2022).

Moreover, bacteriophage‐encoded RNA polymerases (Figure 4) are greatly used as synthetic biology tools and are frequently part of complex genetic circuits. The most classic example is the T7 expression system. This system is based on a lactose‐responsive repressor, lacI and the T7 RNA polymerase. Upon induction with lactose, the repressor stops blocking the expression of the polymerase, which starts driving the expression of the gene of interest (Dubendorf & Studier, 1991). This breakthrough prompted interest in generating T7 RNA polymerase mutant libraries that responded to different stimuli. For example, split polymerase versions were created that respond to temperature and light (Baumschlager et al., 2017; Chee et al., 2022). However, when the T7 RNA polymerase was encoded in *L. plantarum*, its activity was found to be surprisingly underwhelming (Heiss et al., 2016).

RNA polymerases have been identified in the genome of phages infecting *Lactobacillus* species (Gradaschi et al., 2021; Kyrkou et al., 2019). Nevertheless, the specific promoters to which RNA polymerase drives expression are unknown. The identification of those could lead to the potential implementation of RNA polymerases and their specific promoters in genetic circuits in *L. plantarum*. Additionally, other RNA polymerases could be identified using bioinformatic tools such as PHASTEST (PHAge Search Tool with Enhanced Sequence Translation), which identify putative prophages within the genomes of bacteria (Wishart et al., 2023).

Thus, the genomes of phages infecting lactobacilli offer several unexplored parts to achieve efficient and versatile regulation of gene expression in *L. plantarum*.

### Signal peptides driving protein secretion

*Lactiplantibacillus plantarum* is considered a safe and effective host for the recombinant production of enzymes and therapeutic proteins (Wells & Mercenier, 2008). Recombinant lactobacilli species can secrete high titre of protein into the growth medium which can be subjected to further downstream purification processes. Considerable interest has been shown in developing Living Biotherapeutic Products where engineered microbes can be used to deliver drugs in the host environment (Heavey et al., 2022). Successful translation of these products depends on robust yields of secreted proteins which mainly relies on the choice of signal peptides coupled to the protein. A study by and colleagues involving functional analysis of 647 hypothetical proteins of *Lactiplantibacillus plantarum* predicted by bioinformatic tools, revealed 112 transporter protein families and 40 protein families comprised of homologous signal peptides (Davray & Kulkarni, 2023). Mathiesen and colleagues also tested the secretion capability of 76 native signal peptides in *L. plantarum* WCFS1 using staphylococcal nuclease (nucA) as the reporter protein (Mathiesen et al., 2009). Lp_3050 and Lp_2145, endogenous signal peptides were shown to secrete significant amounts of the reporter protein in comparison to its other counterparts. These signal peptides have also shown great promise in secreting recombinant proteins in related *L. plantarum* strains. Signal peptides Lp_0373 and Lp_2145 secreted 13.1 and 8.1 kU of amylase per litre of fermentation broth from *L. plantarum* S21, which was significantly higher than the yields produced using its native signal peptide SP_AmyL (Tran et al., 2021). However, it has also been seen that no single signal peptide can guarantee the maximum secretion of a heterologous protein from *L. plantarum*. Factors like protein hydrophobicity, the overall charge of the protein, the presence of the transmembrane helix and the length of anchoring motifs can significantly affect the secretion efficiency of a signal peptide (Mathiesen et al., 2009).

The greatest bottleneck in the applicability of heterologous protein secretion is the requirement to identify the set of signal peptides that can optimally work for a range of different proteins. So far, this involves experimentally screening multiple signal peptides and selecting the candidate that demonstrates significant protein secretion yield. Standard computational tools like SignalP 6.0 can partially expedite the signal peptide analysis, by suggesting suitable signal peptide combinations and their respective cleavage sites (Teufel et al., 2022). However, there are no generalized correlations identified between protein properties and signal peptide sequences that support strong secretion. To gain such understanding, studies are required that systematically test commonly reported signal peptides with multiple proteins having different sizes, hydrophobicity indices, structural repeats and functional activities. Regarding experimental screening, commonly used methods include detecting the proteins in the cell‐free culture supernatant by Polyacrylamide gel electrophoresis (PAGE), Western blot and mass spectrometry analysis. While these techniques have been effective in many cases, limitations pertaining to the detection limit range (at least ~100 nanograms), complex sample preparation procedure and high temporal requirements reduce the wide‐scale adoption of these methods. One strategy to overcome these limitations involves the fusion of a functional enzyme to the protein of interest and indirectly quantifying its yield by assessing the activity of the fused enzyme in the extracellular growth media. For example, the commonly used reporter protein nucA, which is known to be efficiently secreted from *L. plantarum*, can be fused to the heterologous protein of interest. The secretion of nucA‐fused proteins can be assessed by a well‐characterized agar plate‐based assay in which supernatant or bacterial colonies secreting the nucA‐fused protein create a halo proportional to the amount of secreted protein (Langella & Le Loir, 1999). The main drawback of this method is that the fusion of such enzymes considerably increases the size of the protein to be secreted (nucA M.W ~17 kDa). Additionally, it is often not desired for a secreted protein to harbour an active enzyme, especially for applications like living therapeutics. Another approach to overcome this issue could be the fusion of short peptide tags/protein domains to the protein of interest that is part of split GFP/split Luciferase complementation assays. Such assays involve incomplete versions of GFP or luciferase, which are not fluorescent/luminescent until the complementary peptide unit or protein domain binds to them and completes their structure. These methods are relatively simple and fast (<30 min), providing quantitative results with high sensitivity and requiring simple spectroscopic devices (Knapp et al., 2017; Wang et al., 2021). Such methods can also be automated and have been recently reported with other bacteria for achieving higher throughput screening of signal peptides for secretion (Müller et al., 2022).

### Plasmid retention systems

The most common approach to constructing genetically modified *L. plantarum* has been through plasmid engineering. Extrachromosomal plasmid DNA has allowed the expression of heterologous proteins without requiring tedious methodologies of gene integration into the bacterial genome. The choice of selecting vectors with multiple plasmid copy numbers, diverse antibiotic resistance markers and gene expression systems has increased the ease of genetic circuit construction. However, as with any plasmid‐based engineered system, the presence of an active selective pressure is a prerequisite for maintaining the synthetic gene construct in the microbe (Van Zyl et al., 2019). This creates complexities while trying to use these modified bacteria for product upscaling, drug delivery or recombinant enzyme production. The plasmid retention systems developed in *L. plantarum* are highlighted in the following section, along with suggestions to further expand the current portfolio.

## AUXOTROPHY‐BASED PLASMID RETENTION

Auxotrophy‐based plasmid retention employs the principle of deleting an essential gene from the bacterial genome and reintroducing it into the plasmid vector. The essential protein is crucial for the bacterial host metabolism and without it, the bacteria will be unable to grow and divide. This complementation of the essential gene on the plasmid allows for efficient retention of the heterologous protein without any further external selection pressure. Multiple studies have reported creating auxotrophic strains of *L. plantarum* which have maintained the recombinant plasmid for several generations without compromising on growth and protein production. The most used auxotrophic system encodes for the deletion of the alanine racemase (alr) gene from the bacterial genome to inhibit the D‐alanine biosynthesis pathway. Insufficient production of the D‐alanine amino acid prevents bacterial cell wall formation (Palumbo et al., 2004). Earlier reports have shown that the alr gene‐complemented plasmids can be stably maintained for 200 generations in auxotrophic *L. plantarum* in the absence of any selection pressure (Bron et al., 2002). To revalidate this claim, a pSIP609R vector expressing β‐Galactosidase (β‐Gal) was ligated to the endogenous alr gene and transformed in the Δalr auxotroph of *L. plantarum* WCFS1 (TLG02). Post induction, the recombinant strain was able to show moderate segregational stability for 84 generations, with 17% of the population still harbouring the recombinant plasmid, yielding a net titre of 5 U/mg β‐Gal (Nguyen et al., 2011). This vector was further used to secrete heterologous proteins, chitosanase and β‐mannanase into the growth media at concentrations 79 and 50 kU/L, respectively, in the absence of any external selection pressure (Sak‐Ubol et al., 2016). Paul et al. replaced the antibiotic resistance gene in the pSIP609 vector with the alr gene and encoded the constitutive secretion of the oxalate degrading enzyme (oxdC). A Δalr auxotrophic *L. plantarum* WCFS1 strain harbouring this plasmid was shown to significantly reduce urinary oxalate excretion and calcium oxalate deposition in the kidneys of hyperoxaluric rats (Paul et al., 2018). The deletion of the glucosamine‐6‐phosphate synthase (glmS) gene has also been shown to disrupt the hexosamine biosynthetic pathway and inhibit the N‐acetylglucosamine (GlcNAc) synthesis, which is an essential component of the *L. plantarum* cell wall (Rolain et al., 2012). Vector pSIPH497 was created by replacing the antibiotic‐resistant gene with the P_ldh_ promoter‐driven glmS1 gene and transforming it into the glmS1‐deficient *L. plantarum* WCFS1 strain (NZ5333). The recombinant plasmid was stable for 100 generations without any selection pressure and was able to express mCherry when induced with the AIP for the pSIP system (Chen et al., 2018). In addition to these existing systems, natural auxotrophy of the *L. plantarum* strain 17‐5 (ATCC 8014) towards essential vitamins like biotin can be complemented by the biotin synthase gene responsible for the conversion of desthiobiotin into biotin (Bowman & DeMoll, 1993). Auxotrophic strains targeting the pyrimidine biosynthesis pathway can also be used for retaining plasmids in the bacterial hosts. The deletion of the thymidylate synthase (thyA) gene prevented DNA synthesis and cell division in *L. casei*, and was restored by thyA gene complementation in the plasmid (Zhou et al., 2018). The recombinant strain showed stable retention of the plasmid for over 50 generations and active production of the antimicrobial peptide, lactoferricin. Such strategies can also be extended to *L. plantarum* in the future depending on the intended application. The only major drawback of this promising strategy involves the generation of permanently modified microbes with altered genetic loci. This limits the versatility of plasmid‐based engineering since the plasmids can then only be used in strains that are already genomically modified, restricting the options of testing recombinant plasmids in multiple strains or related genera.

## TOXIN–ANTITOXIN‐BASED PLASMID RETENTION

**FIGURE 5:**
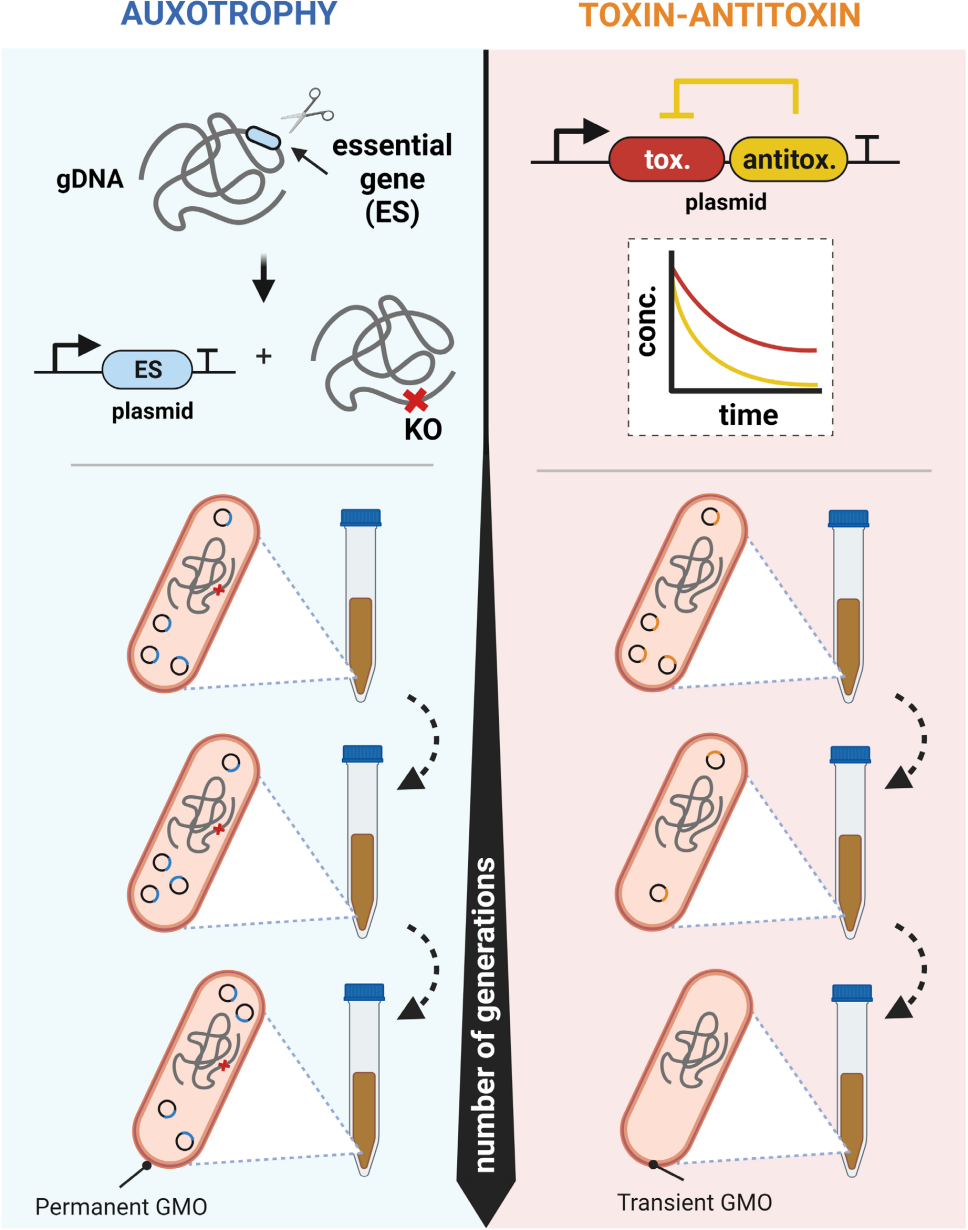
Comparison between the auxotrophy and the toxin–antitoxin‐based plasmid retention approaches.

The adaptive evolution of bacteria under resource‐limited conditions allows for selecting specific genes that provide a survival benefit to the host strain. These genes are primarily acquired from the environment through horizontal gene transfer and transposable elements and are retained in the bacteria as extrachromosomal DNA (Arnold et al., 2022). One of the most prominent ways to naturally retain these elements in the recipient organism includes a toxin–antitoxin (TA) gene pair in the acquired gene segment (Mruk & Kobayashi, 2014). These gene pairs have been classified into five major classes depending on the antitoxin's action mechanism, with the majority of TA pairs belonging to the Type I (antisense RNA antitoxin) or Type II (protein antitoxin) class (Singh et al., 2021). The wide diversity of TA pairs prompted researchers to test them for retaining engineered plasmids in bacteria without any external selection pressure. The low‐copy number p256 plasmid in *L. plantarum* NC7 was seen to have high segregational stability during cellular division (Cosby et al., 1989). Detailed analysis of the p256 plasmid showed that the retention ability was correlated to the presence of the Type II TA system based on pemK (toxin) and pemI (antitoxin; Sørvig, Mathiesen, et al., 2005; Sørvig, Skaugen, et al., 2005). The recombinant plasmid, pLPV100 harbouring the chloramphenicol resistance gene cassette (cat) along with the pemK–pemI gene pair showed 100% plasmid retention after 80 generations in non‐selective media. This TA system showed a higher stability function for the toxin protein than the antitoxin protein, causing a significant decrease in the viability of the bacterial cells that had been cured of the plasmid. This crucial observation resulted in further exploration of potential type II TA candidates in related lactobacilli strains over the last decade. TA candidates have been found to be naturally existing in several cryptic plasmids, for example, the RelE (toxin)–RelB (antitoxin) pair was identified in the cryptic plasmids K25p1, pLU4 and pIR52‐1 in *L. plantarum* K25 (Jiang et al., 2018), *L. reuteri* LU4 (Kim et al., 2017) and *L. helveticus* R0052 respectively (Hagen et al., 2010). Type II toxins like MazE have been annotated both in bacterial genomes (Yan et al., 2012) as well as in mobile plasmids (Abriouel et al., 2019). The prevalence of Type II toxins hinted towards their effective mechanism of action which involves targeting the bacterial RNA population due to its endoribonuclease activity. Fedorec et al. (2019) revalidated the claim showing that the Type II Txe (Toxin)–Axe (Antitoxin) system helped in the plasmid retention in *E. coli* for >30 days in comparison to the Type I hok (toxin)–sok (antitoxin) system. We decided to test the stability function of five different Type II TA systems in *L. plantarum* WCFS1 to achieve stable plasmid retention over multiple generations (Dey et al., 2023). The G50 value (generation number with 50% of plasmid cured population) was highest (~85 generations) for the YafQ (toxin)/DinJ (antitoxin) TA system in comparison to the other TA systems. This G50 was further increased to ~110 generations by combining two TA systems (YafQ/DinJ and MazF/MazE) in the same plasmid. This showed that the presence of multiple toxins targeting the bacterial RNA population can be used to increase the temporal duration of plasmid retention. However, none of the tested TA systems could retain the plasmid beyond ~150 generations. This can be attributed to the gradual development of toxin resistance or upregulation of molecular chaperones to counteract the toxin effect (Mutschler & Meinhart, 2011). This TA system‐based approach is promising for plasmid retention despite such long‐term instability. The results also indicate the opportunity to generate recombinant strains with temporary GMO status since they do not require permanent modification in the bacterial genome like the auxotrophy approach. The bacteria that lose the plasmid over time revert to their non‐GMO probiotic forms, thereby offering the possibility to use such transient GMOs with inbuilt temporal biocontainment for medical or food applications (Figure 5). In addition to the plethora of Type II TA systems available, the focus should also be put on exploring the stability function of Type I TA systems (Fozo et al., 2010). The discovery of the Type I Lpt toxin in the plasmid of cheese ripening bacteria, *Lactobacillus rhamnosus*, demonstrated the key role of the TA system in plasmid segregational stability (Folli et al., 2017). Bioinformatic software like T1TAdb (Tourasse & Darfeuille, 2021) and TADB 2.0 (Xie et al., 2018) can help researchers in faster identification and subsequent stability function testing of Type I and Type II TA systems, respectively, in lactobacilli species. A combinatorial library of these different TA systems could then mediate stable plasmid retention over several generations in *L. plantarum* and other related species.

As an alternative to auxotrophy and TA systems, specific genetic parts from the plasmidome of *L. plantarum strains* could be explored as novel plasmid retention systems. A recent comparative genomic analysis on the plasmidome of 105 *L. plantarum* strains has identified 1141 protein families whose genes are uniquely encoded within plasmids and are not present in the chromosome (Davray & Bawane 2023). Such genes could potentially be promising candidates to positively contribute to plasmid retention in *L. plantarum* when cloned to a different vector. Furthermore, cryptic plasmids often contain highly stable plasmid retention strategies that are host‐dependent and are not yet well understood. Thus, cryptic plasmids can be used as vector backbones themselves in which genes of interest can be encoded and stably retained in the host. Such a strategy has been recently demonstrated in the probiotic *E. coli* Nissle 1917 (Kan et al., 2020).

### Biocontainment modules

Lastly, any genetically altered microbe must be carefully designed to prevent its escape and proliferation into the natural environment. Specific guidelines have been set by regulatory agencies to control the release and disposal of genetically engineered microbes and transgenes post‐application (Wilson, 1993). Abiding by these regulations, there have been several successful attempts to establish kill‐switch‐based genetic circuits in conventional microbial chassis like *E. coli* and *L. lactis*. Kill switches mainly rely on proteins that modulate the growth and survival of the microbe post‐production (Wright et al., 2013). The inducer‐regulated expression of this protein creates non‐permissive growth conditions for the microbe. Although there has been no report of a kill‐switch‐based biocontainment strategy for *L. plantarum*, several candidates can be tested for creating such modules.

**FIGURE 6:**
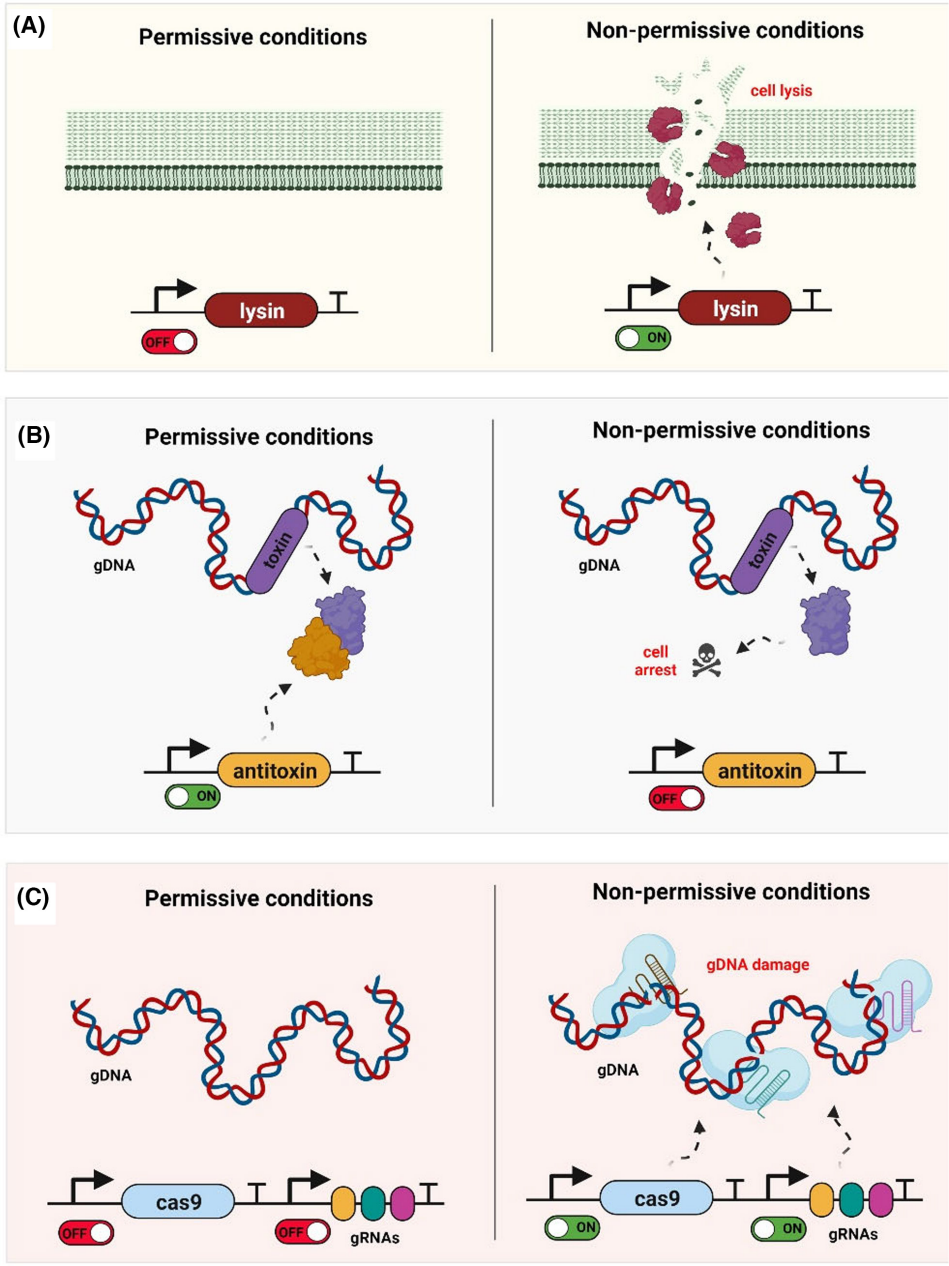
Schematic highlighting the different approaches to constructing kill‐switch genetic circuits in *L.actiplantibacillusplantarum*. (A) Lysin‐based kill switch. In non‐permissive conditions, the expression of the lysin is induced, triggering the cell lysis of the bacteria. (B) Toxin–antitoxin‐based kill switch. In non‐permissive conditions, the expression of the antitoxin is stopped, and the toxin, which is constitutively produced, triggers cell death. (C) CRISPR/Cas9‐based kill switch. In non‐permissive conditions, both the Cas9 and genome‐targeting gRNAs are expressed, which causes the cleavage of the genomic DNA and cell death.

One of the most promising candidates for developing such ‘kill‐switches’ can be the natural (endo)lysins which are an integral part of the bacteriophage genome (Figure 4). When the lytic cycle is induced, lysins are expressed and these enzymes start degrading the bacterial cell wall (Schmelcher et al., 2012). This results in the lysis of the cell and promotes the release of virions outside the bacterium. Despite these fundamental discoveries, not many of these lysins have progressed for translational applications. This provides an opportunity to explore potential kill switch candidates from multiple phages (Figure 6A).

Toxins from orthogonal toxin–antitoxin systems are additional interesting candidates for kill switches in *L. plantarum*. Toxins have been used as part of kill switches in other bacteria. Well‐known examples are the ‘Deadman’ kill switch and the ‘GeneGuard’ system in *E. coli* (Chan et al., 2016; Wright et al., 2015). For *L. plantarum*, an interesting approach could be to integrate the toxin within the genome under a constitutive promoter and then transform these bacteria with a plasmid carrying the antitoxin under an inducible system. Therefore, the expression of the antitoxin would depend on the presence of an inducer (permissive conditions) and without the inducer (non‐permissive conditions), the toxin would trigger cell death (Figure 6B).

CRISPR/Cas9 could also be an attractive alternative to toxic proteins that trigger cell death. The use of CRISPR/Cas9 in a kill switch was successfully employed in the probiotic *E. coli* Nissle 1917, where chemical and temperature‐responsive switches were designed to induce the production of the Cas9 and several guide RNAs targeting different regions of the genome (Rottinghaus et al., 2022). The same approach could be implemented in *L. plantarum* (Figure 6C). Besides, CRISPR has been widely used in *L. plantarum* as a genome editing tool (Myrbråten et al., 2019; Zhou et al., 2019), which strengthens the potential use of CRISPR as a kill switch in these bacteria.

## CONCLUDING REMARKS

As the world is increasingly looking to synthetic biology for solving major global challenges, lactobacilli, with their numerous benefits and ubiquity in our lives, are the ideal chassis to lead this bioengineering revolution. However, the vast diversity within this bacterial family and their poorly understood biochemistry have greatly limited progress in expanding their genetic programmability. Nevertheless, a strong foundation has been laid in a few select species, among which *L. plantarum* is at the forefront. This mini‐review makes the case for establishing *L. plantarum* as a model species among lactobacilli by (i) enumerating the genetic parts established to achieve food‐grade recombinant protein production and (ii) suggesting novel strategies and sources to identify parts that will enable reliable regulation of gene expression and improved biosafety. There are numerous unexplored avenues to expand the genetic toolkit in these bacteria, which have shown promising results in other model organisms like *E. coli* and *B. subtilis*. This knowledge combined with advances in computational tools and automation technologies promises the possibility for rapid development in lactobacilli engineering and the elevation of *L. plantarum* to a model synthetic biology host. In turn, this will expand their applicability in fields such as bioprocess engineering, agritechnology, living therapeutics and engineered living materials.

## AUTHOR CONTRIBUTIONS

**Marc Blanch‐Asensio:** Conceptualization (lead); writing – original draft (lead); writing – review and editing (equal). **Sourik Dey:** Conceptualization (supporting); writing – original draft (equal). **Varun Sai Tadimarri:** Conceptualization (supporting); writing – original draft (supporting). **Shrikrishnan Sankaran:** Conceptualization (equal); writing – original draft (supporting); writing – review and editing (lead).

## CONFLICT OF INTEREST STATEMENT

The authors declare no competing financial interest.
